# Construction Notes

**Published:** 1896-07-04

**Authors:** 


### CONSTRUCTION NOTES.

### New Cottage Hospital at Wells.

The plan of the building is a long-armed T, which was dictated by the shape of the ground. The main doors of approach enter a lobby, whence the inner hall and staircase are approached. The operation room, 15 feet by 12 feet, having a top light, and an adjoining dispensing chamber, open in an eastward direction from an inner hall, and a convalescent room, in a northward direction, whilst the accident ward opens from the hall, as also the sitting-room for matron, in a westward direction. The kitchen, china pantry, linen and store closet, wash-house, larder, coal cellar, and necessary offices communicate with the inner hall from its south side. All these compartments are of ample height. An easy staircase conducts to the upper floor, which consists of a ward for five male patients and a ward for five female patients, each lighted from two sides and one end. The bedroom for the nurse is planned between the two wards, with small windows commanding the whole of each ward for necessary supervision. Bath-rooms and lavatories are reached from each ward through isolating and ventilating lobbies. A ward scullery is arranged conveniently midway between the two wards, with slop-sink, hot and cold water, and a lift for food communicating with the kitchen below. Two other wards, each for one bed, for patients whom it is necessary to isolate, are arranged on the landing of the west side, with the bed-room for the nurse, as on the other floor, placed between the wards, with windows of inspection. The bed-room for servants is on the upper floor. Baths and lavatories are supplied with hot and cold water, and the building is heated with hot water. The hall and operation-room have floors formed of wood blocks. Fresh air is admitted from head height, and the vitiated air is extracted from wards and offices by means of Boyles' air pump ventilators. The wards have also open fireplaces, in addition to the regulated heat from the coils. The architect for the work was Mr. Wilcox, and the builders Messrs. Wibley and Sons, of Bath, whose contract was for £1,718.

### Extension of the Heatherdene Home.

When first the Heatherdene Home was acquired, it was only available for women and children, and the new wing is intended for male convalescents. This has been erected to the south of the original building, which was opened in 1892, and is designed in harmony with the existing building, the entire block now making a very imposing and stately edifice. It consists of three storeys. The lower floor opens out upon the present building with an entrance from the grounds. This floor is devoted to a large day-room facing south-west for the use of male patients; it measures 25 ft. by 17 ft. Adjoining the day-room is the dining-room, having a swing window direct from the kitchen. In immediate connection to the kitchen are the scullery, housemaids' pantry, larder, &c. There is also on this floor a sitting-room for nurses. Opening from the corridor is a large cloak-room with lavatories, &c. The first floor is devoted entirely to sleeping accommodation for male patients; one room with four beds, and another for seven beds. There is also a small bed-room for emergencies, and bath-room, lavatory, with usual conveniences. The second floor has a bed-room for seven beds, and one room with one bed for a male attendant; also a room for servants, which is entered only from the main building. This floor is fitted with the usual lavatory accommodation. The whole of the rooms are warmed with open fires, and are thoroughly ventilated by the admission of cold air through tubes, and the extraction of vitiated air by the aid of heated vertical shafts. The exterior is built entirely of local stone. The contractors for the works have been Messrs. Ives and Co., of Shipley, and the building has been designed and erected under the superintendence of Mr. John Ettringham, architect, Sunderland. The necessary outlay in connection with the extension is as follows: building, £3,025; furnishing, £500; additional land, £1,000.

**Extension of Ulster Eye, Ear, and Throat Hospital.**

The foundation stones of a new wing which is being added to the above hospital have just been laid. This addition will comprise six wards, a special operating room, new and improved sanitary appliances, and two day rooms for men and one for women, as well as a laundry. It will be built the same height as the hospital proper, viz., four storeys. Mr. Isaac Hewitt has been entrusted with the building contract, and Mr. Henry Seaver, C.E., is the architect.

